# Population pharmacokinetics of lopinavir and ritonavir in combination with rifampicin-based antitubercular treatment in HIV-infected children

**DOI:** 10.1186/1758-2652-13-S4-O24

**Published:** 2010-11-08

**Authors:** C Zhang, P Denti, USH Simonsson, G Maartens, MO Karlsson, H McIlleron

**Affiliations:** 1 University of Cape Town, Cape Town, South Africa; 2 University of Cape Town, Cape Town, South Africa; 3 University of Uppsala, Uppsala, Sweden

## Objectives

Children with HIV associated tuberculosis often require co-formulated lopinavir/ritonavir (LPV/RTV)-based antiretroviral treatment with rifampicin-based antitubercular treatment (ATT). Rifampicin (RIF), a potent inducer of drug-metabolizing systems, profoundly reduces the bioavailability of LPV. The aims of this study were to develop an integrated population pharmacokinetic (PK) model describing LPV and RTV PK in children with and without concomitant ATT using two different dosing approaches and to estimate doses of LPV/RTV achieving target exposures during ATT in young children.

## Methods

A population PK analysis was conducted in NONMEM. During ATT 15 children were given LPV with extra RTV (LPV/RTV ratio 1:1) and 20 children were given twice the usual dose of LPV/RTV (ratio 4:1) 12 hourly; 39 children without tuberculosis and 11 children undergoing repeated sampling after ATT were treated with standard 12 hourly doses of LPV/RTV (median LPV dose 11.6 mg/kg). Goodness-of-fit plots and visual predictive checks were used to evaluate the models.

## Results

In a one-compartment model with first-order absorption to describe LPV PK, and a one-compartment model with transit absorption for RTV, the dynamic influence of RTV concentration on the clearance of LPV was modelled as direct inhibition with an Emax model. Allometric scaling for weight was used for clearance and volume of both LPV and RTV. During ATT, the relative oral bioavailability of LPV was reduced by 79% in children receiving twice the usual dose of LPV/RTV. The clearance of RTV was 18 L/h with, and 13 L/h without, ATT. The baseline clearance of LPV, when RTV was undetected, estimated 4.34 L/h. With increasing concentrations of RTV, clearance of LPV decreased in a sigmoid relationship (EC50 0.051 mg/L). Volume of distribution for LPV and RTV were 11.7 and 102 L, respectively. Simulations predicted that children weighing 4-6, 6-8, 8-12 and 12-18 kg need respective doses of 65, 50, 37 and 30 mg/kg LPV/RTV (4:1) 8 hourly in order to maintain LPV concentrations > 1 mg/L in at least 95% of children.

## Conclusions

The model describes the drug-drug interaction between LPV, RTV and RIF. Using 8 hourly doses, approximately 2.5 to 5.5 times the standard doses are required to maintain therapeutic LPV concentrations in young children during ATT.
